# ^18^F-FDG PET/CT Findings in Acute Epstein-Barr Virus Infection Mimicking Malignant Lymphoma

**DOI:** 10.3390/diagnostics6020018

**Published:** 2016-05-12

**Authors:** Mathilde Ørbæk, Jesper Graff, Elena Markova, Gitte Kronborg, Anne-Mette Lebech

**Affiliations:** 1 Department of Infectious Diseases, Copenhagen University Hospital Hvidovre, Kettegaards Allé 30, DK-2650 Hvidovre, Denmark; mathilde.jensen@sund.ku.dk (M.Ø.); gitte.kronborg@regionh.dk (G.K.); 2 Department of Clinical Physiology & Nuclear Medicine, Copenhagen University Hospital, DK-2650 Hvidovre, Denmark; jesper.graff@regionh.dk; 3 Department of Radiology, Copenhagen University Hospital Hvidovre, DK-2650 Hvidovre, Denmark; gitte.kronborg@regionh.dk

**Keywords:** Epstein-Barr virus, ^18^F-FDG PET/CT, lymphadenopathy, infectious mononucleosis, malignant lymphoma

## Abstract

We present a case demonstrating the diagnostic work-up and follow-up of a patient with acute Epstein-Barr virus (EBV) infection in which the clinical picture and imaging on ^18^F-FDG PET/CT mimicked malignant lymphoma. Follow-up ^18^F-FDG PET/CT scan in the patient performed 7 weeks after the abnormal scan revealed complete resolution of the metabolically active disease in the neck, axillas, lung hili, and spleen. This case highlights inflammation as one of the most well established false positives when interpreting ^18^F-FDG PET/CT scans.

**Figure 1:**
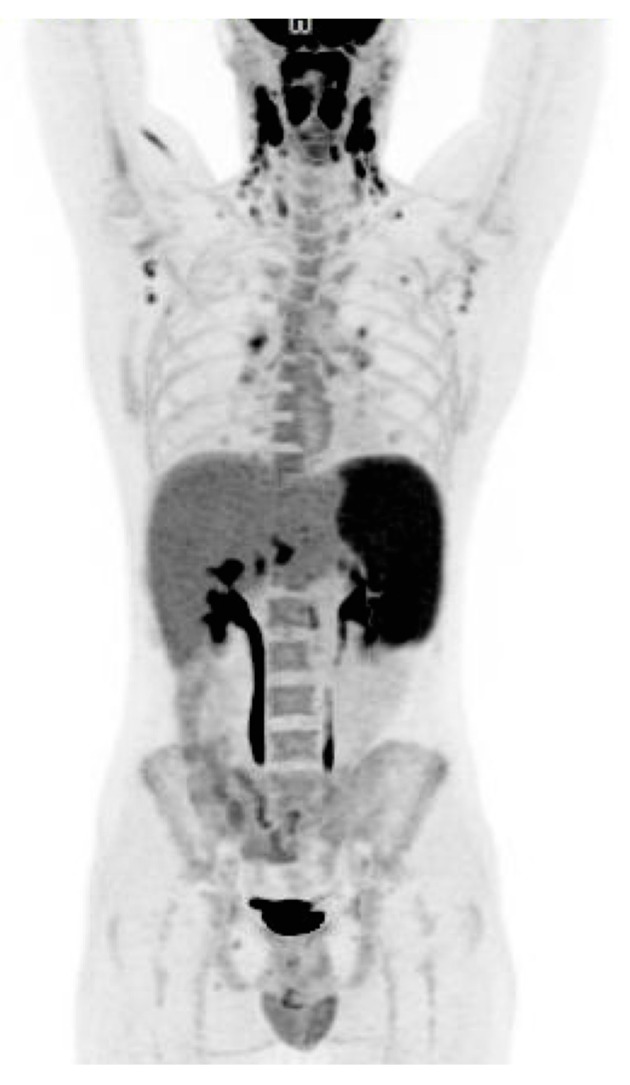
A 21-year-old man presented with 6 weeks of unexplained fever, pharyngitis, enlarged cervical neck lymph nodes, night sweats and severe fatigue. The patient was treated as an outpatient with antibiotics for acute tonsillitis twice without any improvement. He had persistent daily fever and a 6 kg unintentional weight loss. No significant past medical history. Epstein-Barr virus (EBV) and Cytomegalovirus (CMV) titers drawn one week after debut of symptoms were negative. Three months before presenting with symptoms the patient had been travelling in South East Asia for 3.5 months. He had not been sick during his travel abroad. Physical examination was significant for lymphadenopathy at the cervical neck. No splenomegaly was noted. At admission white blood cells was 13.0 × 10^9^/L (normal range: 3.5–8.8 × 10^9^/L) with 10.0 × 10^9^/L lymphocytes (normal range: 1.0–3.5 × 10^9^/L). Alanine aminotransferase (ALT) was elevated at 104 U/L (normal range: 10–70 U/L) and Lactate dehydrogenase (LDH) was elevated at 939 U/L (normal range: 105–205 U/L). Due to concern of malignant lymphoma an ^18^F-FDG PET/CT was performed (Figure 1). The scan revealed pathologically ^18^F-FDG uptake in enlarged lymph nodes in the bilateral regions of neck, axillas, lung hili, and in an enlarged spleen (18 cm). Findings were suspicious for malignant lymphoma. A lymph node biopsy was considered.

**Figure 2:**
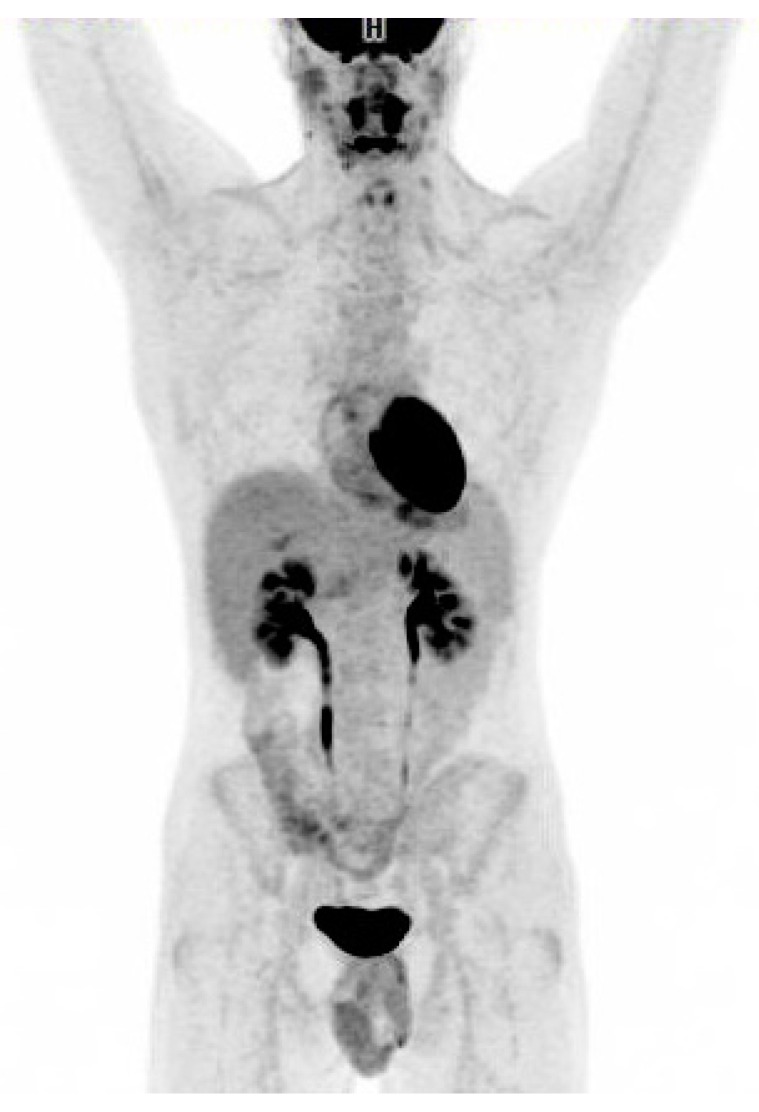
Additional diagnostic workup drawn a day prior to the ^18^F-FDG PET/CT became available showing positive EBV VCA-IgM and negative EBV VCA-IgG indicating acute infectious mononucleosis. CMV-IgM was furthermore positive. HIV and hepatitis panels were negative. Given the patient’s age, the clinical course with no past medical history and acute EBV titers, the patient’s abnormal ^18^F-FDG PET/CT scan was most likely secondary to acute EBV infection and no lymph node biopsy was performed. The patient was closely monitored. After a week he had resolution of symptoms clinically, ALT had increased to 461 U/L, LDH to 780 U/L. Twenty days after the initial ^18^F-FDG PET/CT scan the patient was feeling well, he had regained his appetite and returned to daily activities although still complained of tiredness. ALT = 193 U/L, LDH = 295 U/L. At this time both EBV VCA-IgG and EBV VCA-IgM antibodies were positive. CMV IgG was negative and CMV-IgM was borderline indicating a previous false positive CMV-IgM probably induced by the EBV infection. Follow-up ^18^F-FDG PET/CT scan in the patient performed 7 weeks after the initial scan (Figure 2) revealed complete metabolic response. Except for one lymph node which had decreased in size from 2.0 cm to 1.2 cm, all the other lymph nodes had regressed to normal size on CT. The size of the spleen had normalized (14 cm). ALT and LDH were normal. Reporting of this case is in accordance with the ethical standards of the institutional and/or national research committee. ^18^F-FDG uptake in EBV infection has been reported previously in a few cases in children [1] and organ transplant recipients [2]. Only two cases of ^18^F-FDG uptake in acute adult EBV infection have previously been reported [3,4]. In conclusion, this case illustrates acute adult EBV infection in a non-immunocompromised adult with EBV-induced lymphadenopathy that mimicked malignant lymphoma on ^18^F-FDG PET/CT scan. It highlights inflammation as one of the most well established causes of falsely interpreting ^18^F-FDG PET/CT as suspicious of malignancy. Any inflammatory process can demonstrate hypermetabolic activity because of the high degree of glucose metabolism by activated cells of inflammation [5,6]. As always, the benefits of ^18^F-FDG PET/CT in diagnostic work-up should be viewed in light of the radiation burden to the patient.
